# Fréquence et caractéristiques des AVC impliquant les artères perforantes dans le Service de Neurologie de l’Hopital Bafelatanana, Antananarivo

**DOI:** 10.11604/pamj.2017.28.76.13593

**Published:** 2017-09-26

**Authors:** Nomena Finiavana Rasaholiarison, Rahamefy Odilon Randrianasolo, Lala Andriamasinavalona Rajaonarison, Jenny Larissa Rakotomanana, Julien Razafimahefa, Alain Djacoba Tehindrazanarivelo

**Affiliations:** 1 Service de Neurologie, Hôpital Befelatanana, Antananarivo, Madagascar

**Keywords:** Accident vasculaire cérébral, artères perforantes, neurologie Hôpital Befelatanana, récurrence, troubles cognitifs, Stroke, perforating arteries, department of Neurology, Befelatanana hospital, recurrence, cognitive disorders

## Abstract

**Introduction:**

Les accidents vasculaires cérébraux des artères perforantes sont surtout des artériolopathies. Ils évoluent vers la démence et la récurrence. Pour mieux prévenir ces complications notre étude avait pour but d'évaluer la fréquence et les caractéristiques de ces AVC.

**Méthodes:**

c'est une étude descriptive rétrospective du 01 Mars au 25 Septembre 2015 au service de Neurologie CHU-JRB. Ont été inclus les patients présentant un déficit neurologique brutal et un scanner cérébral avec atteinte du territoire profonde. Les caractéristiques des AVC des artères perforantes ont été recueillies. Les données étaient traitées par le logiciel SPSS 20.

**Résultats:**

Quatre-vingt-trois (48,25%) patients avaient des AVC des artères perforantes sur 172 AVC. Pour les AVC des artères perforantes la population était jeune avec 65,06% moins de 65 ans et à prédominance masculine avec 61,44%. Les formes hémorragiques étaient à 67,46%. Trente et un patients (37,34%) ont connu des récurrences et parmi eux presque le quart avait 2 récurrences avec 38,70% en moins de un an. Tous les patients avec récurrence avaient un trouble dysexécutif (p < 0,0001) et une mauvaise observance thérapeutique d'antihypertenseur. La mortalité n'était qu'à 6,02% pour ces types d'AVC pendant l'hospitalisation.

**Conclusion:**

Un suivi spécifique en neurologie est nécessaire dès le premier accident vasculaire cérébral d'artère perforante pour dépister un début de démence et prévenir les récurrences.

## Abstract (en)

**Introduction:**

Strokes of the perforating arteries are mainly arteriolopathies. They result in dementia and stroke recurrence. This study aimed to evaluate the frequency and characteristics of these strokes to better prevent these complications.

**Methods:**

We conducted a descriptive, retrospective study in the department of neurology at the Befelatanana general hospital, Antananarivo over the period 01 March-25 September 2015. All patients with abrupt neurological deficit and deep brain involvement on brain scanner were included in the study. The features of strokes involving the perforating arteries were collected. Data were processed with SPSS 20 software.

**Results:**

Out of 172 patients with a stroke, 83(48.25%) had stroke involving the perforating arteries. Stroke involving the perforating arteries affected young people (65.06%) aged less than 65 years and preferentially the male population (61.44%). Haemorrhagic forms accounted for 67.46%. Thirty-one patients (37.34%) had stroke recurrences and, among them, almost a quarter had 2 recurrences (38.70%) in less than a year. All patients with recurrence had dysexecutive disorder (p < 0.0001) and poor antihypertensive medication adherence. Mortality accounted for only 6.02% in patients with onset of these strokes during hospitalization.

**Conclusion:**

Specific neurologic follow-up is necessary after a first stroke involving perforating arteries in order to make an early diagnosis of dementia and to prevent recurrences.

## Introduction

Les accidents vasculaires cérébraux (AVC) des artères perforantes sont les déficits neurologiques brutaux d'origine vasculaire présumés atteignant les branches profondes des artères cérébrales. Ce sont surtout des artériolopathies. Ils se manifestent par les Infarctus lacunaires (obstruction des artérioles) ou des Hémorragies intra-parenchymateuses sous corticales (rupture des petits vaisseaux dont les parois sont nécrosées) [1]. Ils évoluent vers la démence (subcortical vascular cognitive impairement ou troubles cognitifs sous corticaux d'origine vasculaire) et la récurrence [2]. De démence à cause de leurs localisations en sous corticales où se trouves les différentes connexions du cerveau et de récurrence car ce sont des maladies des petites artères qui sont déjà présents. Pour mieux prévenir la survenue de ces complications, notre étude avait pour but d'évaluer la fréquence et les caractéristiques des AVC des artères perforantes.

## Méthodes

Une étude rétrospective longitudinale du 01 Mars au 25 Septembre 2015 était menée auprès du service de Neurologie CHU-JRB. Nous avons inclus les patients présentant un déficit neurologique brutal avec atteinte du territoire profond au scanner cérébral. Nous n'avons pas inclus les patients avec AVC des artères superficielles et les patients sans scanner cérébral. Nous avons exclus les dossiers incomplets.

Les caractéristiques socio-démographiques, cliniques et paracliniques des patients inclus ont été recueillies. Pour ces caractéristiques, l'âge de survenu est considéré comme précoce si le patient a moins de 60 ans, le genre : masculin et féminin, le type de l'AVC: ischémique ou hémorragique selon le scanner cérébral, la sévérité par le score NIHSS ou National Institutes of Health Stroke Scale (inférieur à 14 ou non), le profil évolutif de l'accident unique ou récurrent. Un AVC est considéré comme récurrent s'il suit l'une des conditions suivantes: un déficit neurologique différent de l'AVC index (le premier AVC), un accident impliquant un territoire vasculaire ou anatomique différent de l'AVC index, un accident concernant un autre type d'AVC que l'AVC index. Nous avons mesuré : la prévalence globale des AVC tout confondu parmi les patients hospitalisés, la prévalence spécifique des AVC des artères perforantes parmi tous les AVC, le taux de mortalité spécifique par AVC des artères perforantes durant l'hospitalisation. Nous avons aussi étudié les caractéristiques de la récurrence : leur nombre, les intervalles des récurrences, l'existence de troubles dysexécutifs évalué par le score de BREF (Batterie Rapide d'Efficience Frontale) et la régularité du traitements antihypertenseur chez ces patients selon leurs antécédents. Nous avons analysés la corrélation de ces différents facteurs avec la récurrence. Les données étaient traitées sur le logiciel SPSS 20.

## Résultats

Sur 357 hospitalisés dans le service de Neurologie pendant cette période 157 avaient des accidents vasculaires cérébraux. Donnant une prévalence globale de 48,17% (172/357) de notre échantillon. Sur ces 157 patients 83 avaient un AVC des artères perforantes soit 48,25%. L'âge moyen de notre population d'étude était de 59,09 ans dont 59,03% avaient moins de 60 ans. Elle avait une prédominance masculine avec 61,44% de cas. Plus de la moitié de ces AVC des artères perforantes étaient hémorragique avec 67,46% ; mais ils avaient un bon pronostic car 92,77% de ces patients avaient un score NIHSS inférieur à 14 (**Tableau 1**). La mortalité par ce genre d'AVC était de 6,02% (**Tableau 1**).

**Tableau 1:** Les caractéristiques socio-démographiques de la population

Caractéristiques	N	%
**Age (moyen: 59,09 ans)**		
< 60 ans	**49**	**59,03**
≥ 60 ans	34	40,96
**Genre**		
Masculin	**51**	**61,44**
Féminin	32	38,55
**Type**		
Hémorragique	**56**	**67,46**
Ischémique	27	32,83
**FDRCV**		
HTA	**70**	**84,33**
Diabète	3	3,61
Tabac	23	27,11
Alcool	30	36,14
**Complications**		
Pneumopathie	**12**	**14,45**
Escarre	8	9,6
TVP	0	0
Veinite	0	0
Infection Urinaire	0	0
**NIHSS**		
< 14	**77**	**92,77**
≥ 14	6	7,23

FDRCV: facteurs de risque cardio-vasculaire; HTA: hypertension artérielle; TVP: Thrombose Veineuse Profonde; NIHSS: National Institute Health Stroke Scale

Parmi les AVC des artères perforantes, 37,34 % soit 31/83 faisaient des récidives, dont 80,64% (25/31) une fois et 19,35% en faisait plus de 2 fois. La récurrence survenait après 1an dans 61,29% (19/31) des cas. Tous ces patients présentaient des troubles dysexécutifs avec un test de BREF inférieur à 15. Un antécédent de traitement antihypertenseur irrégulier était noté dans 98,7% des cas. Nous avons trouvé une association de cette récidive avec certains facteurs comme l'âge avec p = 0.024, le NIHSS avec p = 0.016 et les troubles dysexécutifs avec p < 0.0001 (**Tableau 2**). La durée d'hospitalisation était moins de 7 jours.

**Tableau 2:** Association de la récurrence et les facteurs pouvant l’influencer

Facteurs	TOTAL 83	Récurrents 31	Non récurrents 52	p
**Age <60 ans**	48 (57.83%)	13 (41.93%)	35 (67.30%)	**0.024**
Genre masculin	49 (59.03%)	21 (67.74%)	28 (53.84%)	0.213
Type hémorragique	53 (63.85%)	19 (61.29%)	34 (65.38%)	0.707
**NIHS < 14**	77 (92.77%)	26 (83.87%)	51 (98.07%)	**0.016**
**Score de BREF < 15**	31 (37.34%)	31 (100%)	0	**<0.0001**
Antihypertenseur irrégulier	82 (98.79%)	30 (98, 7%)	52 (100%)	0.193

BREF: Batterie Rapide d’Efficience Frontale ; NIHSS : National Institute Health Stroke Scale

## Discussion

Les AVC des artères perforantes sont aussi importants car ils représentaient la moitié de tous les AVC. Ils étaient hémorragiques dans 67,46% des cas. Ils étaient négligés car 37,34% d'entre eux étaient récurrents et tous les patients ont eu un traitement anti-hypertenseur irrégulier. Ils atteignaient les sujets jeunes moins de 60 ans dans 59,03 des cas. Ces AVC étaient peu mortels avec un taux de mortalité à 6,02% durant l'hospitalisation. Mais ils étaient handicapants surtout sur le plan cognitif car en effet tous les patients présentant une récurrence avaient un trouble dysexécutif. Nous avons trouvé une association de cette récidive avec l'âge des patients, le score NIHSS et les troubles dysexécutifs. C'est le premier article existant à Madagascar qui traite à propos des artères perforantes cérébrales, leur récidive et les troubles cognitifs qu'ils peuvent engendrer. Ils existent peu d'article récent qui traitent isolément les artères perforantes et les récurrences. Cela nous permet de voir un autre type d'handicap que physique, le handicap cognitif. Cependant notre étude a ses limites, nous avons une faible taille d'échantillon donc notre étude est peu représentative. L'étude a été faite seulement au sein du service de Neurologie avec une période d'étude limitée et un absence de suivi. L'estimation de la démence n'a été faite qu'au niveau des patients qui présentent des récurrences et par le score de BREF alors qu'au cours de leur évolution tous les AVC peuvent donner des démences.

Nous avons eu 48,25% d'AVC des artères perforantes, Jong G et al en 2007 en Hollande a trouvé la même prévalence avec 57 % [3]. Laloux P et al ont trouvé 19% [4] car dans son étude comme dans les autres pays développés, les AVC des artères perforantes par artériolopathie ne sont plus courantes. Chez eux ce sont surtout les problèmes cardio-embolique et athéroscléreux qui prédominent [5]. L'âge moyen de notre échantillon était de 59,09 ans avec une prédominance masculine dans 51% des cas. Dans les autres études comme celui de Sun Y et al en 1994 en Singapour, de Burn J et al en 1994 en Grande Bretagne, de Benjamin P et al en 2014 aux Etats-Unis, ont eu une population plus vieille avec respectivement un âge moyen de 73 ans, de 72 ans, de 70 ans. La prédominance masculine était de 54%, 54% et 78% pour les 3 auteurs [6, 7,8]. Pour les types, les AVC hémorragiques représentaient 67,46% contre 10% pour Lammie A et al en 2000 en Grande Bretagne mais 75% pour l'étude de Behrouz R et al [1,5]. Et Silva D et al a trouvé 40% d'AVC lacunaire [9]. Cette haute fréquence du type hémorragique pourrait s'expliquer par le fait qu'aujourd'hui comme observer dans cette étude, les patients ont une très faible observance thérapeutique en matière d'hypertension artérielle. La mortalité était seulement de 6,02% dans notre étude contre 17% pour Burn J et al en 1994 en Grande Bretagne et 70% pour Stefano P et al en 1995 en Italie [6, 8]. Nous avons constaté que notre étude avait une faible mortalité par rapport aux autres car les autres études ont été faites 20 ans auparavant et qu'elles concernaient tous les AVC confondu c'est-à-dire superficiels et profonds. Mais la mortalité dans la récurrence double par rapport au premier AVC selon Jerrgensen HS et al [9].

Ces AVC profonds aussi récidivaient. Nous avons observé que 37, 34% récidivaient. Et parmi eux 38,70% ont eu au moins une récidive en moins de 1 an. La récidive a une association avec l'âge et le score NIHSS d'entrée. Pour les autres études, la récidive en moins de 1 an est de 22,66% pour Burn J et al. en 1994 en Grande Bretagne et 30% Passero S et al. en 1995 en Italie [6,10]. Mais ces études concernent tous les AVC confondus. Jerrgensen HS et al ont trouvé une récurrence de 23% même après contrôle des traitements de prévention de récidive [11]. Pour Laloux P et al 32% de sa population récidivait dont 61% en moins de 1 an [4]. Pour Behrouz R et al 7,7% de récurrence en moins de 1an [5]. L'étude de Moroney JT et al a montré que ce sont surtout les gros vaisseaux qui récidivaient car chez eux en ce moment ils prédominent et que les petites artères ne récidivent que dans 1% [12]. Et Silva D et al a 1/3 de récurrence [10]. Pour Sun Y et al. 47% de récidive dont 30% en moins de 1 an. Pour Gonzalez-Duarte et al. 36% récidive la première année [7]. Il a eu 2,1% en 1 an et a aussi observé que la récidive peut être hémorragique même si l'index était ischémique et vice versa [13]. Mais récurrence s'accompagne souvent de démence car il existe une augmentation des dommages cérébraux selon Pendlebury ST dans son étude. Ils ont trouvé que 41,3% dans ses récurrences étaient déments et que ce sont des syndromes frontal plutôt que des troubles de mémorisation [14]. Dans la littérature les troubles cognitifs sous corticaux d'origine vasculaire précoce (< 65ans), sont surtout des artériolopathies, touchent la substance blanche et s'expriment surtout par des troubles dysexécutifs [2]. Dans notre échantillon, tous avaient un trouble dysexécutif. C'est-à-dire un score de BREF inférieur à 15 avec une association de celle-ci avec la récidive. Mais dans celui de Leys et al en 2005 en GB [15], ils en avaient 13,3% en utilisant le score CDRS IV. Comme le principal facteur de risque pour la lésion des artères perforantes est l'hypertension artérielle nous avons pu constater que 98,7% de notre population étaient hypertendus avec traitement irrégulier alors que c'est moins marqué dans l'étude de Stefano P et al en 1995 en Italie avec 47% [10]. Et que les maladies des petites artères et Accidents Vasculaires Cérébraux par hypertension artérielle se trouve toujours en territoires profonds. Pour Laloux P et al. 79% de son échantillon étaient hypertendus et 64% étaient non observants [4]. Pour Gonzalez-Duarte et al. 86% étaient hypertendus dont 56% non contrôlés [12]. Selon Hanger HC et al, le contrôle de l'hypertension artérielle diminue les récidives [15], pourtant nous n'avons pas trouvé d'association entre récidive et traitement irrégulier.

Dans notre étude nous avons trouvé une prévalence élevé des AVC concernant les artères perforantes. Ils touchent surtout les sujets jeunes. Ils sont hémorragiques, récurrents et négligés. Ils donnent un handicap physique mais aussi un lourd handicap cognitif. Nous avons observé que notre population avait le même profil de patient victime d'AVC qu'il y a 20 ans dans la littérature. Cela a permis ainsi de nous cadrer sur le suivi de nos patients en post-AVC et leur observance thérapeutique. Nous n'avons pas étudié l'impact de ces troubles cognitif sur l'efficacité de la rééducation physique. Une étude sur l'influence de l'état cognitif et la rééducation physique est encore à faire. Mais aussi une étude comparative entre récurrence et démence avec des Accidents Vasculaires Cérébraux des artères superficielles.

## Conclusion

Dans notre étude les Accidents Vasculaires Cérébraux impliquant les artères perforantes touchaient surtout les hommes jeunes, souvent hémorragiques intra-parenchymateux. Ils étaient peu mortels. Quand ils récidivaient, ces AVC étaient handicapants à court terme sur le plan cognitif. Nous émettons alors qu'un suivi spécifique en neurologie est nécessaire dès le premier AVC d'artère perforante pour dépister un début de démence et prévenir les récurrences. Ainsi qu'un suivi en cardiologie car le contrôle de la tension artérielle peuvent contribuer à prévenir un second AVC. Plusieurs études seront nécessaires pour déterminer la réalité à Madagascar, sur les étiologies, la prévention des récurrences et la survenue de démence, ainsi que la rééducation de ces patients.

### Etat des connaissances actuelle sur le sujet

- Les troubles cognitifs et atteinte des petites artères;
- Les rôles de l'HTA et des facteurs de risque cardio-vasculaires dans l'atteinte des petites artères profondes cérébrales.

### Contribution de notre étude à la connaissance

- Il spécifie les atteintes des petites artères et ses relations avec les troubles cognitifs dans un pays africain où les contrôles des facteurs de risque sont encore mineurs;
- Il apporte une précision sur le rôle de l'AVC dans les troubles cognitifs surtout en Afrique où l'aspect cognitif en tant qu'handicap est négligé;
- Il permet une réflexion sur la rééducation en post-AVC qui sera difficile, à cause troubles cognitifs qui va s'installer.

## Conflits d’intérêts

Les auteurs ne déclarent aucun conflit d'intérêt.
